# Wireless Ultra-Low-Power Sensor Platform for Environmental Monitoring

**DOI:** 10.3390/s25247486

**Published:** 2025-12-09

**Authors:** Jannis Winnefeld, Metin Kizilarslan, Werner Knop, Jens Passoke

**Affiliations:** Fakultät 1-Elektro-und Informationstechnik, Hochschule Hannover, 30459 Hannover, Germany; jannis.winnefeld@hs-hannover.de (J.W.); werner.knop@hs-hannover.de (W.K.); jens.passoke@hs-hannover.de (J.P.)

**Keywords:** IoT, wireless energy, energy efficient electronics, battery-free sensor platform

## Abstract

This paper presents an open, modular sensor platform based on wireless energy and data transmission. The platform is powered by the carrier signal of a transceiver and transmits the measured sensor data using backscatter modulation. Through the use of modular ready-to-buy components, the sensor platform can be flexibly adapted to different applications and is therefore suitable for both building automation systems and industrial automation tasks. Energy storage, power management, and modulation are designed so that the overall energy demand of the platform is mainly determined by the sensor in use. The performance of the system was verified with a demonstrator measuring underfloor temperature and humidity. The demonstrator operates at a carrier frequency of 868 MHz, an output power of 27 dBm EIRP at the transceiver antenna, and an antenna gain of 0 dBi at the receiver antenna. A transmission range of more than 3 m has been achieved. The platform provides an input sensitivity of −15 dBm. Its open design enables a straightforward scaling from prototype to small- and medium-volume production.

## 1. Introduction

The increasing use of IoT devices in building automation and industrial applications highlights the growing importance of simple and flexible methods for integrating sensor technologies. Sensors can measure environmental data such as temperature, humidity, and presence, enabling automated, demand-driven control of technical systems such as heating and air conditioning. This offers substantial energy-saving potential, particularly in the building sector, where studies have shown that automated, occupancy-based control of heating, ventilation, and air conditioning (HVAC) systems can reduce energy consumption by up to 45% [1]. Similar potential can be found in industrial applications, for example in the continuous monitoring of plant conditions, temperatures, material parameters, or gas concentrations. However, the use of conventional sensors is often accompanied with special challenges: Many relevant measuring points are located in areas that are difficult to access manually. In such cases, retrofitting or adapting a conventional sensor system can be problematic due to the necessary power supply. Either the cabling requires considerable installation effort, or the periodic replacement of batteries increases maintenance costs and material consumption. Consequently, battery-free sensor solutions are gaining importance [2].

However, solutions that meet both the requirements of accessibility to hard-to-reach measuring points and flexible adaptability to different applications are inherently limited. To illustrate these limitations and to determine the necessary requirements for such a sensor system, a specific use case is considered. In this paper, a sensor installed under the floor and designed to measure temperature and humidity serves as an example. For the use case described here, an energy harvesting solution that relies on temperature differences, light or other electromagnetic sources to power the sensor platform is unsuitable. Such a device must operate reliably in various locations within a building where a consistent energy supply from ambient sources, such as ambient RF energy, cannot be guaranteed [3]. A targeted energy supply via electromagnetic waves, on the other hand, offers a number of advantages over other approaches and enables battery-free operation. A well-established technology that has applied this principle for more than 20 years is UHF RFID, standardized under ISO 18000-6C [4]. Here, an identification medium (transponder) is identified via a carrier signal of a transmitter (reader) while data transmission is carried out by backscatter modulation. The combination of such a transponder with sensor technology therefore offers a proven basis for a battery-free sensor solution. Another argument in favor of this solution is that the cost of readers is falling steadily thanks to the high level of component integration. Sensor tags available on the market that use this technology are generally limited to specific applications and feature integrated, non-exchangeable sensors. The TELID 412 [5] is an example of a device designed exclusively for temperature sensing. Consequently, it is not suitable for the use case described here, which additionally requires humidity sensing, or for other application scenarios requiring different sensing capabilities. Platforms with open interfaces that allow free selection of external sensors have disappeared from the market.

Nevertheless, several sensor platforms reported in the literature could serve as reference designs or models. For instance, ref. [6] describes a sensor platform that can be operated at a reception power of −17 dBm and supports the connection of various sensors. However, it is based on the Monza X-2K, which, like other models of the same type, is no longer available. In [7], another open sensor platform is presented that allows various sensors to be connected via interfaces. Experimental investigations with a standard RFID reader show that the platform is only activated at a reception power of at least −5.9 dBm. As a result, and due to legal limits on transmitter power, the achievable range is low, and reliable operation at shielded measuring points cannot be guaranteed.

Based on these considerations, this work aims to develop an open, modular sensor platform that incorporates the key advantages of UHF RFID systems for sensor applications. These include, in particular, battery-free power supply via the carrier signal of a transmitter and energy-efficient data transmission using backscatter modulation. Due to the lack of ISO 18000-6C transponders with sensor interfaces, the implementation is based on commercially available discrete components. Various sensors can be integrated via digital interfaces such as I^2^C, allowing the platform to be flexibly adapted to different application scenarios. To present the platform, a demonstrator is used that is designed for the described application scenario of temperature and humidity measurement under the floor. The development is based on the regulations for European short-range devices (SRD). Accordingly, it operates at the frequency range around 868 MHz with a maximum transmission power of 0.5 W (27 dBm). A transceiver transmits the energy required to operate the demonstrator and receives the data.

This article is structured as follows: The next section first outlines the framework conditions of the sensor platform before describing how the individual components work using the demonstrator as an example. Section 4 follows with the experimental validation of the platform, in which key data such as sensitivity and range are determined. Finally, Section 5 discusses the platform concept and addresses possible extensions and improvements.

## 2. Sensor Platform Requirements

The sensor platform is based on the design of passive UHF RFID transponders for energy transfer and communication. The maximum permissible transmission power of the transceiver depends on the specific classification of the system. If the sensor platform operates as a general short-range device up to 0.5 W (27 dBm) is typically permitted. If classified as an RFID system, up to 2 W (33 dBm) will be allowed [8]. However, this transmission power may only be used with directional transmitter antennas. Because of the position of the transceiver on the wall and the demonstrator beneath the floor, a maximum radiation angle of $70^{\circ }$ must be maintained. This limitation would require the use of larger antennas. Therefore, the system is designed to operate at a transmission power of 0.5 W (27 dBm). The sensor platform should achieve a minimum range of 3 m. Under free-space conditions and assuming an antenna gain of 0 dBi the received power can be conservatively estimated to be −15 dBm or 32 µW. Within an estimated measurement interval of 10 min, the sensor platform should take less than a minute to measure and transmit the data, which corresponds in a duty cycle of 10%, as required by SRD regulations.

In order to minimize the computing power of the sensor platform, the aim is to connect the sensor via a digital interface. Compared to conventional RFID transponder systems designed in accordance to ISO 18000-6C, this implementation offers several simplifications: The sensor platform does not need to receive any commands initially. Communication is used exclusively for the transmission of measurement data and unique identification. In the demonstrator example, temperature and humidity are recorded and transmitted in conjunction with a 64-bit ID and the necessary header and padding bits using FM0 coding. Data transmission occurs once the sensor platform has received sufficient energy from the electromagnetic field. Therefore, no data logging is not required. This also eliminates the need for a battery. Only buffer capacitors are used to store enough energy to then start data transmission. Anti-collision mechanisms are also omitted, as it is assumed that only one sensor is located in the area of a transceiver.

The aim is to achieve a compact footprint of 3 cm × 3 cm for the sensor platform in order to minimize installation costs in scenarios such as underfloor deployments. FR4 is used as the circuit board material to enable cost-efficient manufacturing of the platform. For the same reason, as many functional groups as possible are to implemented using integrated circuits available on the market. The demonstrator is intended to show that the above-described goals of the sensor platform can be achieved and that energy and data transmission is possible even in difficult installation environments.

## 3. Architecture of the Sensor Platform

**Figure 1:**
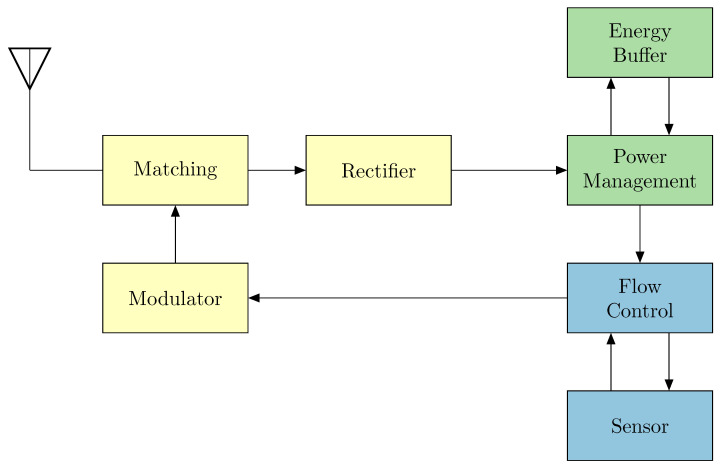
Architecture of the sensor platform: Front end with matching network, rectifier, and modulator (yellow)—Energy processing with energy storage and power management (green)—Sensor section consisting of flow control and sensor (blue).

The architecture of the sensor platform is shown in Figure 1 as a block diagram.

In this chapter, the requirements for the individual components are defined, and their selection for implementation is described.

### 3.1. Flow Control

The function of the flow control is to read out the current sensor data when sufficient energy is available, i.e., after the power-on reset, and to transmit the data together with the ID and other data security bits to the modulator. In RFID transponders, this is usually implemented via a hard-wired flow control. The flow control does not require significant computing resources, therefore the primary focus is on low energy consumption. On the sensor platform, a microcontroller is to take over the task of flow control in order to provide flexibility in interfacing with different sensors. A similar approach has been described in [7], among others. The functional scope of the microcontroller and its integrated peripherals can be reduced to a minimum, allowing the focus to be placed on energy efficiency. Microcontrollers that operate in the threshold or subthreshold range, which were introduced a few years ago by companies such as Plsense, e-peas, ST, and Renesas, would be particularly suitable here. However, there limited availability, insufficient performance for the requirements of the sensor platform, and lack of development environments have let to a focus on already established ultra-low-power microcontrollers. Studies such as [9,10] analyze such microcontrollers in terms of their minimum operating voltage and average power consumption. Most of these microcontrollers operate at a supply voltage of 1.8 V or higher. For example, the MSP430 series from Texas Instruments requires a quiescent current of 42 nA in low-power mode, which corresponds to an energy consumption of 75.6 nW, while significantly higher values are required during active operation, when reading the sensor and transmitting the data. The MSP430F1232 microcontroller consumes around 470 µA at a clock frequency of 3 MHz, which corresponds to a power consumption of 0.85 mW at 1.8 V.

Ultimately, the ATtiny43U (Microchip Technology, Chandler, AZ, USA) [11] was chosen as the processor, which operates at an operating voltage of 1.8 V or higher and has an operating current of approximately 30 µA with an internal RCO at 128 kHz. The maximum transmission rate is determined by the clock rate. We deliberately avoid using special function blocks of the processor to ensure straightforward migration to other microcontrollers. Furthermore, the chosen clock frequency represents a practical compromise between transmission time and energy consumption. As with UHF RFID transponders, data is transmitted to the modulator using FM0 encoding. Each half-bit requires 10 processor cycles. This results in a transmission rate of approximately 6.4 kBaud. However, the receiver must take into account that the RCO can fluctuate depending on the production lot, temperature, and operating voltage.

The SHT40 from Sensirion (Staefa, Switzerland) [12] was chosen as the sensor for the demonstrator because it operates at a supply voltage of 1.1 V or higher, and its low power consumption allows it to be switched on directly via a processor port. Other sensors, such as the BMxxx series from Bosch Sensortec (Reutlingen, Germany) [13] or inertial sensors such as the LSM6DSO from STMicroelectronics (Geneva, Switzerland) [14], can also be controlled via the I^2^C or SPI with the processor. The requirements for the sensor are that it operates at an operating voltage of 1.8 V or higher and is as energy-efficient as possible, and that the interface does not require a minimum clock rate.

### 3.2. Power Management and Energy Storage

The received carrier signal must be converted into a usable voltage via a rectifier, for which, as already mentioned in Section 2, −15 dBm or 32 µW are available. Even if an ideal rectifier were used (as is theoretically possible [15]), the energy obtained would not be sufficient to directly operate the selected ATtiny43U microcontroller. In practice, this problem is exacerbated because even efficient front ends, such as those described in [16], only achieve around 50% efficiency with an input power of −15 dBm and an optimal load resistance of 20 kΩ. Assuming a conservative efficiency of 25%, approximately 8 µW is available at the output of the rectifier, which corresponds to an output voltage of only about 280 mV. This results in several requirements:

1. The output voltage at the rectifier is significantly lower than the operating voltage of 1.8 V. In order to operate typical flow control elements such as the ATtiny43U, the voltage must therefore be increased. This can be achieved either by using multi-stage rectifier cascades or by using special power management circuits with integrated boost converter. However, both options are associated with additional efficiency losses.
2. Even if the required operating voltage is reached, the available current is not sufficient to operate the flow control element over a transmission cycle.
3. Since both the load resistance and the input power can vary greatly, the circuit should have a Maximum Power Point Tracker (MPPT).

Integrated power management circuits from the field of energy harvesting, such as those offered by e-peas, Analog Devices, or EM-Microelectronic, meet these requirements. These components already provide functions such as a boost converter, MPPT, and a power-on reset (POR) for the added circuitry. However, most of these solutions are designed for specific energy sources such as solar cells or mechanical sensors and are therefore not matched to the power and voltage available at the output of the rectifier. In the MEH [3] project, power management circuits for RF energy harvesting have already been evaluated. The best results were achieved with circuits from e-peas. Now there are a large number of derivatives of the AEM family. The AEM30300 from e-peas (Louvain-La-Neuve, Belgium) [17] was chosen for the sensor platform. With a minimum input voltage of 275 mV and a minimum energy of 6 µW at the input (or 3 µW if there is still residual energy in the storage), the component can be supplied with the conservatively estimated efficiency of a rectifier and charge a buffer storage, thus providing sufficient energy for the flow control circuit and the sensor.

To activate the POR, the internal circuitry monitors the voltage at the storage and switches an output pin at certain voltage levels. The switching thresholds of the hysteresis circuit can be set to specified configurations via the external circuitry. These configurations are designed for storage media such as lithium batteries or supercapacitors. This input signal can be used to control switches such as the TMUX1101 (Texas Instruments, Dallas, TX, USA) [18], which switch the voltage and reset the flow control. Different switching thresholds for activation ($V_{\mathrm{on}}$) and deactivation ($V_{\mathrm{off}}$) prevent the TMUX from entering an unstable state. As a further option, the TPS3840PL from Texas Instrument (Dallas, TX, USA) [19], an energy-efficient reset device, was considered for this task.

Ceramic capacitors are used as storage elements because they are compact, are not subject to charging cycle limitations, and react only slightly to temperature fluctuations. The desired duty cycle between charging and data transfer can be controlled via the capacity used, together with the hysteresis of the POR circuit. The storage element must be designed to provide sufficient energy for operating the microcontroller and sensor, while ensuring that the charging time remains short enough to activate the sensor platform.

### 3.3. Front-End

**Figure 2:**
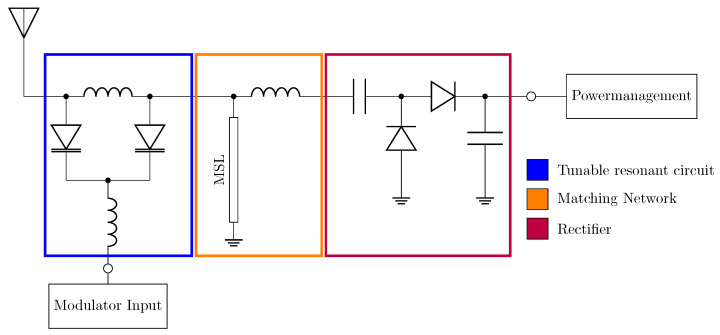
Schematic of the front end compromising a tunable resonant circuit (blue), a matching network (orange) with a microstrip line (MSL), and a rectifier (red). The antenna port is matched to 50 Ω.

The final block is the front end, which consists of a rectifier, a matching circuit, and a modulator. The design used is based on the circuit presented in [20] and is shown in Figure 2.

The front end has a single-stage rectifier and achieves a conversion efficiency of 30% at an input power of −15 dBm with a load resistance of 10 kΩ. At an input power of 0 dBm, the efficiency increases to 51%. These values are lower than those reported in [21,22], which is expected since, in the design used here, the modulator is integrated into the matching network. This configuration introduces additional losses due to the parasitic effects and limited quality factor of the varactor diodes used. Although this reduces the achievable efficiency, the resulting performance remains very good for the targeted input power range.

Multi-stage rectifiers, such as those used in [7], are not suitable for the considered use case. As described in [23,24], the optimum efficiency is achieved with one to two stages. Although additional stages can achieve a higher output voltage, the overall efficiency decreases significantly, especially at low input power and low load.

By choosing the AEM30300 as the power management circuit, the voltage at the output of the rectifier is sufficient and no further stages are required to generate a higher voltage. The choice of load resistor is influenced by the power management system, whose efficiency also depends on this load. Therefore, a compromise must be found between the efficiency of the rectifier and the power management system. The efficiencies achieved are below the values of 50% listed in [16] for the same input power. However, this is due to the fact that polyimide is used as the substrate instead of FR4 and a load resistance of 20 kΩ is used. However, this load resistance would reduce the efficiency of the power management system and thus the overall efficiency.

An adjustable parallel resonant circuit is used as the modulator, which is implemented with SMV1248 varactor diodes from Skyworks (Irvine, CA, USA) [25]. In [20], the parallel circuit was compared with a series circuit, and it was shown that the parallel resonant circuit has better characteristics. The modulator is energy-efficient, as only the charging currents of the varactor diode contribute to the energy requirement. The resonant circuit is connected to the matching network between the antenna and the rectifier, with the antenna port matched to 50 Ω. Applying a reverse voltage through the flow control element (port of the ATtiny43U) shifts the resonance frequency of the resonant circuit, which changes the matching of the antenna. The reflection factor of the front end can be switched between $|S_{11}|=0.158$ (absorption) and $|S_{11}|=0.738$ (reflection), thus enabling modulation of the carrier.

Compared to other front ends, such as those described in [26] or [27], SPDT switches or MOSFETs are commonly used as modulators. However, internal investigations show that such components typically exhibit quiescent currents in the µA range. The resulting continuous energy losses can discharge the energy storage faster than it can recharge at the available input powers. This would reduce the overall efficiency more than a backscatter module integrated in the matching network.

Less than 20 nA is required for the backscatter switching process using the approach described in Figure 2. With an input power of −15 dBm, the sensor platform would consume 9.5 µW. This demonstrates that the combined design of rectifier, matching network, and modulator achieves an exceptionally low overall energy consumption.

## 4. Sensor Platform Demonstrator: Floor Sensor

**Figure 3:**
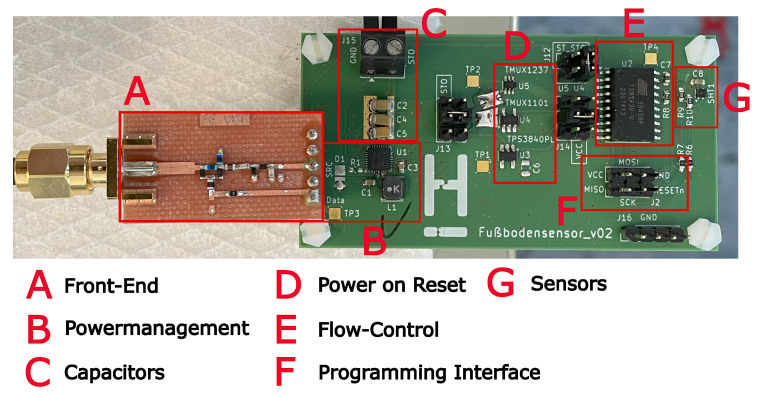
Demonstrator with individual modules.

Figure 3 shows the sensor platform demonstrator. The previously selected components in Section 3 are used here. The properties of the individual assemblies will be described before discussing the overall performance.

The design shown in Figure 3 is not optimized for minimum dimensions in terms of size, and the front end is built on a separate circuit board. The reason for this is that several measuring points and configurations (e.g., for the AEM30300) are required for validation, and additional options (e.g., for the POR) had to be tested with the demonstrator. All components are placed on FR4 substrate.

### 4.1. Energy Consumption of the Flow Control and the Sensor

**Figure 4:**
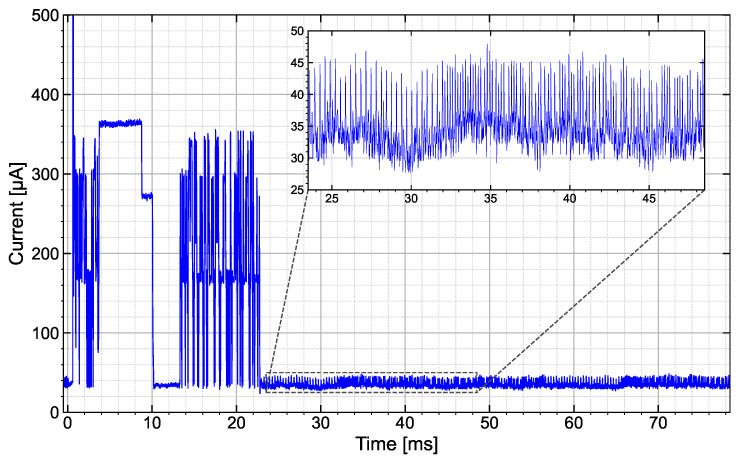
Power consumption of the sensor and flow control during measurement, readout, and data transmission to the modulator. The inset shows the power consumption during data transmission to the modulator.

The energy requirements of the microcontroller and the sensor are decisive for the charging time and required buffer capacity. Therefore, in a first step, the current was measured in a time-resolved manner to determine the duration and power consumption of the individual phases of the program sequence, as shown in Table 1. Figure 4 shows the measured current in µA at a fixed supply voltage of 1.8 V. The individual phases of the program sequence are given in Table 1:

The energy required for reading the sensor data, including three complete data transfers (26 bytes each) to the modulator, amounts to approximately 18 µJ. Of this, approximately 12 µJ is required for the sensor to respond and read out the data and for the internal determination of the sensor data.

The energy requirement is therefore mainly determined by the sensor, especially since the data sets transmitted to the modulator are highly redundant due to triple transmission. The pull-up resistors on the data and clock lines are the determining factors in data transmission between the sensor and microcontroller via the I^2^C interface. Tests have shown that the resistance should not be significantly higher than 10 kΩ (data sheet specification) for the sensor to function properly. For the demonstrator, 13.2 kΩ is used to keep the current as low as possible while still ensuring proper functioning.

When determining sensor data (temperature and humidity measurements) using the SHT40, energy consumption can be minimized by reducing the accuracy of the measurements. The highest accuracy was selected for the demonstrator, resulting in a read time of around 8.2 ms and a maximum sensor current of 310 µA. The operating current of the microcontroller is approximately 30 µA. This corresponds to the data sheet specifications for a 1.8 V supply voltage and a clock rate of 128 kHz using the internal RCO. In the enlarged segment of Figure 4, individual current peaks reaching a maximum of 50 µA can be seen, which are caused by the capacitance change of the modulator’s varactor diodes. It should also be noted that switching on the SHT40 via the microcontroller port, a current peak of 4 mA occurs. This behavior is due to a 100 nF buffer capacitor on the sensor’s power supply.

The energy balance of flow control and sensor can be used to calculate the approximate buffer capacity required. Assuming a voltage hysteresis of 0.3 V for the POR circuit (see Section 3.2), the calculated 18 µJ results in a capacity of

(1) $$C_{\mathrm{Buff}}=2\cdot W/{\left(\Delta U\right)}^{2}\approx 400\;\mu F.$$

### 4.2. Power-on-Reset and Power Management

First, the TPS3840PL20 Nano Power Voltage Supervisor reset module was considered. An increase in operating voltage causes an increase in the operating current of the circuit and a longer charging time. According to the ATtiny43U data sheet [11], an increase of 0.5 V in operating voltage results in an increase of approximately 10 µA in operating current. This has a significant effect on the energy balance. For this reason, the operating range of the TPS3840 was selected so that it is close to the minimum operating voltage of the ATtiny43U. The switch-on voltage is 2.1 V and the switch-off voltage is 2.0 V. In order to read the sensor and send two data sets with this voltage hysteresis, a buffer capacitor of theoretically 3600 µF is required. This relatively large capacitor also results in a long charging time when the capacitors are completely discharged. Furthermore, the quiescent current of the TPS3840 is around 300 nA. This means that a 300 µF capacitor discharges at about 1 mV/s. After 10 min during which the transmitter does not transfer any energy to the sensor, the voltage at the capacitor has discharged from 2 V to 1.5 V.

In contrast, the two components TMUX1101 [18] and TMUX1237 from Texas Instruments (Dallas, TX, USA) [28] have a quiescent current of approximately 3 nA, which results in significantly lower discharge of the buffer capacitors. The TMUX1101 is a low-leakage-current (single-pole single-throw) precision switch and the TMUX1237 is a general-purpose switch (single-pole double-throw). Measurements have shown that the properties of the two components do not differ significantly for this application, so only the TMUX1101 will be considered in the following.

For controlling the POR switch, an output of the power management IC AEM30300 can be used. This output monitors the voltage of the buffer capacitor and supports different types of storage elements through discretely configurable voltage thresholds. The setting for Solid State Battery [17] has proven to be suitable here. This activates the ST_STO output of the AEM30300 when 2.3 V is reached and switches off again when the voltage falls below 2.0 V. This means that the hysteresis is 0.3 V and the switch-on voltage is around 0.2 V higher than that of the TPS3840. Although this results in an increased operating current for the processor, the significantly lower quiescent current compensates for this disadvantage.

Alternatively, the AEM30300 allows the switching thresholds, i.e., the hysteresis, to be individually set to 1.8 V–2.0 V using four external resistors connected to the AEM30300. However, the resistors form a voltage divider that may have a maximum resistance of 100 MΩ. At a storage voltage of 2 V, this divider would draw a continuous current of 20 nA from the capacitors. Furthermore, the tolerances of the resistors have a significant influence on the hysteresis values. For this reason, this option was not implemented in the demonstrator.

The AEM30300 features maximum power point tracking (MPPT). In the configuration used here, the open-circuit voltage is measured for 5.2 ms at a cycle time of 280 ms, and the load voltage is set to be half the open-circuit voltage for the next cycle. The cyclic switching of the load causes modulation interference, therefore, using a fixed 47 kΩ resistor as a reference for the load was tested as an alternative. The resistor is designed for the worst-case scenario (−15 dBm input power), as there is no dynamic adjustment to the available input power. No significant deterioration in power management was observed with the fixed resistor. However, dynamic MPPT is used for further considerations.

Based on an input power of −15 dBm at the front end, a buffer capacity of 300 µF, and the AEM30300 configurations described above, measurements were performed to determine the demonstrator’s response time to an energy supply from an external source. The measurement data are listed in Table 2.

The table shows that the time until the first data transfer is approximately the same for both POR modules. However, assuming the sensor data is to be retrieved every 10 min, the TMUX1101 only requires 25 s of supply time, compared to 90 s for the TPS3840. This shows that the TMUX is significantly more energy-efficient due to its lower quiescent current. The TPS3840’s low power-on time of 50 ms is also notable; no complete data sets can be transferred during this time after reading the SHT40. This is due to the low hysteresis of the TPS3840 of 0.1 V.

For these reasons, the TMUX1101 and the AEM30300, configured to use a voltage ranging from 2.0 V to 2.3 V, are used for all further measurements.

### 4.3. Demodulator/Front-End

The characteristics of the front end (rectifier and modulator) have already been described in [20]. Therefore, only a brief discussion of the combination of the front end with power management and modulation characteristics will be provided here.

**Figure 5:**
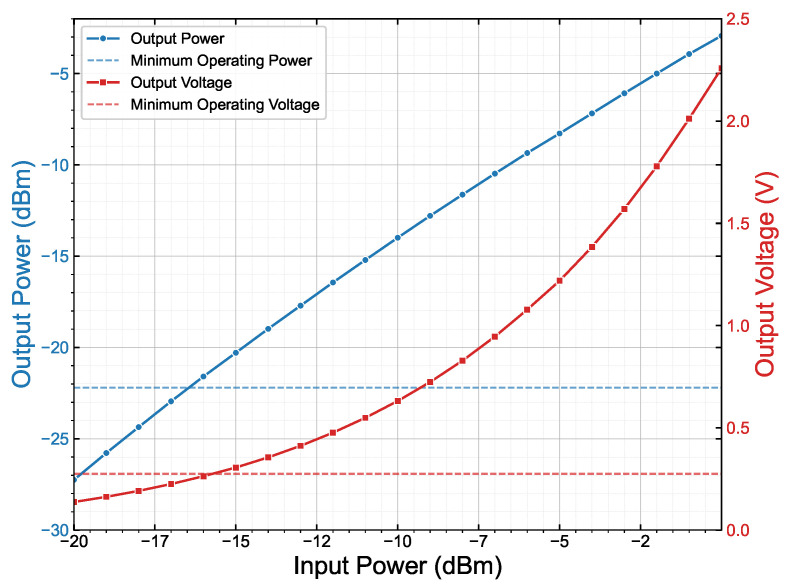
Output power (blue) and output voltage (red) as a function of front-end input power. The horizontal lines indicate the minimum input power and input voltage of the AEM30300.

Figure 5 shows the output power and output voltage of the front end as a function of the input power. A fixed 10 kΩ resistor was used as the load. The figure also shows the response thresholds of the AEM30300, i.e., minimum input power and the minimum input voltage. This shows the assumed −15 dBm at the input of the front end at a range of 3 m are above the response thresholds.

Due to the varactor diodes used in the modulation branch, the modulation depth respectively reflection voltage is also determined by the operating voltage. Depending on the choice of POR component, the voltage is between 2.0 V and 2.3 V and thus above the minimum 1.8 V, supporting the function of the modulator. If the modulator is not activated, as much of the energy as possible should be used to operate the circuit. This results in a reflection factor of

(2) $$\Gamma_{i}n\left(U=0V\right)=0.1486\cdot e^{j\cdot 153^{\circ }}$$

When driven by a voltage of 2 V, the reflection coefficient becomes

(3) $$\Gamma_{i}n\left(U=2V\right)=0.785\cdot e^{j\cdot 2.3^{\circ }}$$

### 4.4. Overall Performance of the Demonstrator

The properties of the demonstrator are now to be tested in a real environment to validate its suitability as a floor sensor for building management. Small low gain antennas are used for this purpose. The reasons are as follows:

(a) To test the assumptions made above about the range (where the antenna gain was assumed to be 0 dBi).
(b) When installed underfloor, the sensor platform should require as little space as possible (e.g., 60 mm × 60 mm × 20 mm).

The last requirement rules out an antenna with high gain. This again applies to the transceiver, as it should be similar in size to a room control unit for heating.

The test setup uses two quadrifilar antennas (circularly polarized) from Caen (WANT020) [29], each realizing a 0.2 dBi gain. The antennas are optimally aligned to each other.

Although laboratory equipment (generator, spectrum analyzer, or SDR) could also be used for measuring, a transceiver board and accompanying software was developed for the iHKV (integrierter Heizkreisverteiler) project (to be published), funded under grant number 03EN1065B. This board will be described in a forthcoming publication.

The transceiver board is operated with a 867.5 MHz CW output power of 27 dBm at the antenna port. The backscattered signal is filtered and amplified via a directional coupler, mixed down to baseband via an IQ demodulator, digitized via AD converters, and evaluated by software. Due to the monostatic design, a carrier cancellation circuit is also integrated, that minimizes the coupled carrier signal before each measurement.

**Figure 6:**
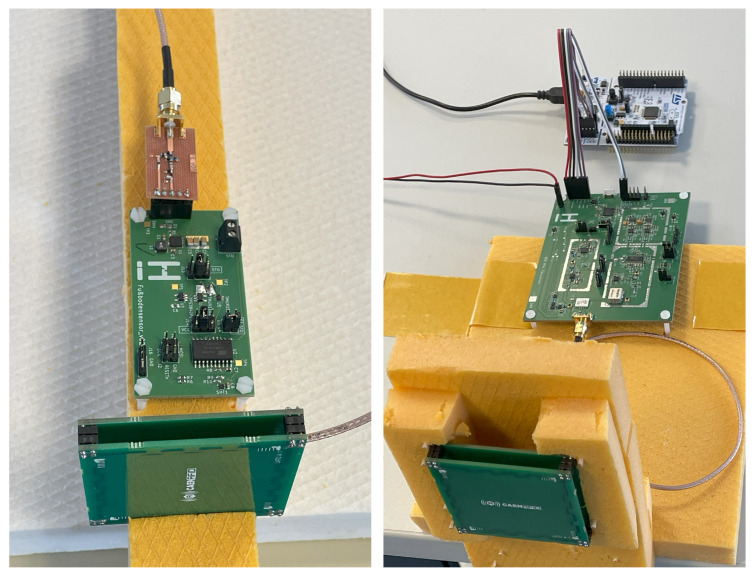
Antenna with demonstrator board (**left**) and receiving antenna with transceiver board and communication board (**right**).

Figure 6 shows the test setup. The measurements were performed in a laboratory environment. Here, multipath propagation can occur, resulting in a constructive and destructive interference. Therefore, the received power at the sensor antenna was first measured in a distance of 0.5 m up to 4 m between the transmitting and receiving antennas. At a distance of 1.7 m, there was a strong destructive interference, i.e., the reception power was −17 dBm. Otherwise, the reception level was always above −15 dBm, aligning closely with the theoretical curve according to the Friis equation. At a distance of 2.8 m, the level was −15 dBm. Therefore, the duty cycle and reception characteristics were analyzed at this distance. It was found that with the capacitor charged and a field-off-time of 10 min, at least one measurement was taken after approximately 30 s of activation time and three data packets were received.

**Figure 7:**
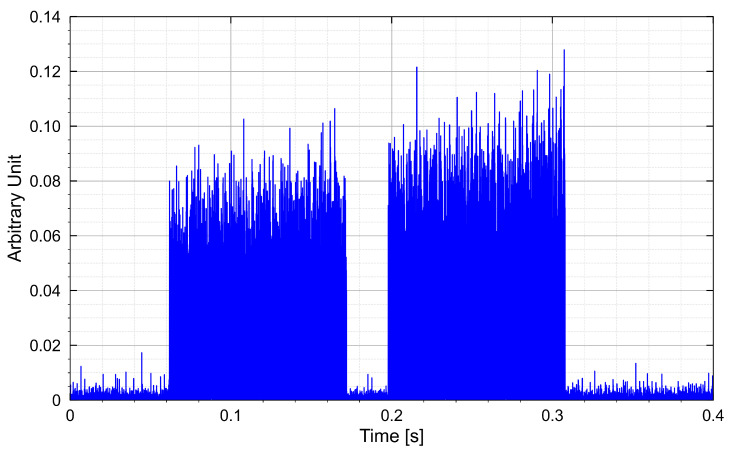

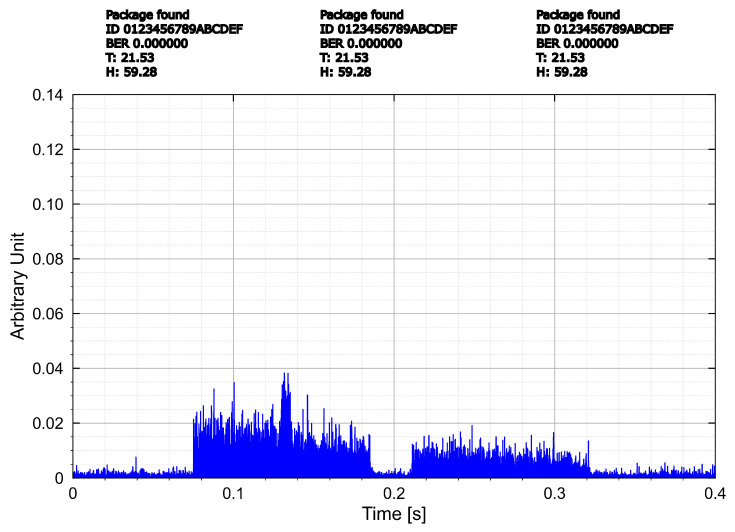
Measured and processed data at a distance of 3 m (**top**) and 3.7 m (**bottom**). In between: decoded received data for 3 m.

If the distance between the antennas is increased to 3.7 m, the demonstrator is still supplied with −17 dBm. Here, too, the sensor platform could still be activated, but only after an activation time of 60 s. In most cases, only one valid data packet was received. Figure 7 shows the received data before evaluation and the decoded data for a distance of 3 m. At 3.7 m (bottom diagram), data can still be received. However, it often contains bit errors because of a high noise level in the modulation. The signal-to-noise ratio is 21 dB for a distance of 3 m and 7.5 dB for 3.7 m.

These measurements validate the range and transmission quality of the demonstrator under real-world conditions.

## 5. Summary and Outlook

A passive sensor platform was designed that enables contactless readout of sensor data over a distance of at least 3 m and operates with an input power of less than −15 dBm at 868 MHz. The platform’s functionality was validated using a room monitoring demonstrator measuring humidity and temperature. Here, a range of more than 3 m was achieved with a transmission power of 27 dBm. The sensor platform is powered by the field, and the energy is stored in ceramic capacitors until activation. The POR circuit reduced the discharge of the capacitors by 3 nA by using the TMUX1101. This results in a short activation time for the demonstrator from the field energy provided by the transmitter. The processor for flow control runs at a low clock rate of 128 kHz. This results in a working current of approximately 30 µA at 1.8 V for the processor. Data transmission via backscatter demodulator with varactor diodes does not significantly increase the current either. The required energy for the platform is significantly influenced by the sensor used, which in the demonstrator set up requires approximately two-thirds of the total energy for sensor determination and data transmission to the microcontroller.

Further energy savings are possible by lowering measurement accuracy or reducing the number of transmitted data sets. The processor’s operating current is a particularly decisive factor in energy consumption. The ATtiny43U used in this work could, for example, be replaced by the STM32L011k4 from STMicroelectronics (Geneava, Switzerland) [30], which would reduce the operating current to 20 µA. However, in order to significantly increase the read range of the sensor platform, either the efficiency of the front end has to be improved or a power management circuit with higher sensitivity is required. A comparison with other publications on rectifier circuits for RF power harvesting shows that an efficiency of 30% at −15 dBm input is already an outstanding value. To our knowledge, there is no power management circuit with a lower switch-on threshold available on the market. An upstream boost converter could help to achieve the switch-on threshold even at low voltages [3], but this would reduce the efficiency.

The sensor platform has a flexible design thanks to the use of standard components, allowing other or additional sensors to be integrated and read contactlessly over a relatively large distance. The use of a microcontroller makes it straight-forward to integrate the sensors into the flow control. It is important to ensure that the sensors are as energy-efficient as possible and operate at a supply voltage of 1.8 V or lower. However, this requirement excludes only a few sensors. For example, climate sensors such as the BME280 and BME688 from Bosch Sensortec (Reutlingen, Germany) [31,32], or the previously mentioned inertial sensor LSM6DSOX (STMicoelectronics, Geneva, Switzerland) [14], also meet these requirements.

Thus, the presented approach enables the development of a family of contactless and battery-free sensor technologies suitable for both building automation and industrial applications.

## Figures and Tables

**Table 1:** Energy consumption and time progression during sensor data acquisition and transmission.

Time [ms]	Energy [µJ]	Program Step
1	0.06	Initialize microcontroller
3	1.00	Activate Sensor
9	6.00	Sensor data acquisition
10	4.00	Read sensor data
3 × 34	3 × 2.3	Transfer data to modulator

**Table 2:** Comparison of activation times and operating cycles of the TPS3840 and TMUX1101 components.

	TPS3840	TMUX1101
Activation time (fully discharged)	210 s	210 s
Activation time (after 10 min without power supply)	90 s	25 s
Power on	50 ms	150 ms
Transmission cycle (continuous supply)	35 s	14 s

## Data Availability

The original contributions presented in this study are included in this article and its Supplementary Materials. Further inquiries can be directed to the corresponding authors.

## Supplementary Materials

The following supporting information can be downloaded at: https://www.mdpi.com/article/10.3390/s25247486/s1, Raw data from which Figure 4, Figure 5 and Figure 7 was generated.
