# Supplementary figures and images for: Wireless Ultra-Low-Power Sensor Platform for Environmental Monitoring
Source: Sensors (Basel). 2025 Dec 9;25(24):7486. doi: 10.3390/s25247486 (PMC12737270; doi:10.3390/s25247486)

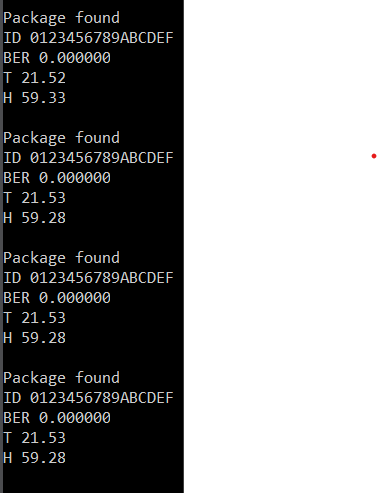

Supplement: Supplementary file 1 [file sensors-25-07486-s001.zip › Datasets_MDPI_Publication/data_package_figure7.png]
